# Imaging of Artificial Tumor Models in an Anatomical Breast Phantom with a Single-Sided Magnetic Particle Imaging Scanner

**DOI:** 10.3390/tomography12050060

**Published:** 2026-04-24

**Authors:** Christopher McDonough, John Chrisekos, Matthew Jurj, Alycen Wiacek, Alexey Tonyushkin

**Affiliations:** 1 Physics Department, Oakland University, Rochester, MI 48309, USAtonyushkin@oakland.edu (A.T.); 2 Center for Biomedical Research, Oakland University, Rochester, MI 48309, USA; 3 Department of Electrical and Computer Engineering, Oakland University, Rochester, MI 48309, USA; 4 Department of Bioengineering, Oakland University, Rochester, MI 48309, USA

**Keywords:** MPI, magnetic particle imaging, breast cancer, imaging, nanoparticles, SPIO

## Abstract

**Background:** Magnetic Particle Imaging (MPI) is an emerging biomedical imaging modality that detects superparamagnetic iron oxide nanoparticles (SPIONs), providing high contrast, sensitivity, and quantification capabilities without ionizing radiation, making it particularly suitable for cancer diagnostics. Considerable engineering efforts are underway to translate MPI technology to clinical settings. Most of these MPI scanners feature a cylindrical bore geometry similar to that of other clinical imaging modalities, which limits their potential application primarily to head scanning. **Methods:** We have developed a single-sided MPI scanner designed to expand the modality’s applicability to other regions of the human body through a unique hardware design developed in our previous work. Imaging experiments were performed on an anatomical breast phantom containing implanted SPION point sources placed at anatomically plausible locations for breast tumors. These point sources served as artificial tumors for evaluating the system’s suitability for breast imaging applications. **Results:** The scanner successfully detected and clearly resolved the implanted SPION tumors in two orthogonal imaging planes. Tumor positioning was independently validated by ultrasound imaging, confirming MPI’s accurate localization. In addition, sensitivity measurements demonstrated a detection limit of 4.0 μg of iron, below the estimated 4.8 μg sensitivity threshold required for breast tumor detection with electronic depth scanning up to 3.5 cm deep. **Conclusions:** Together, these results demonstrate the capability of a single-sided MPI geometry for breast imaging applications. Imaging an anatomical breast-shaped volume presents significant challenges for MPI due to the size and accessibility constraints of conventional hardware. The results presented highlight the advantages of this approach and support its potential to extend MPI from small-animal imaging to clinically relevant applications.

## Simple Summary

Magnetic Particle Imaging (MPI) is a new imaging technique that detects iron oxide nanoparticles with high sensitivity and no radiation, making it promising for cancer detection. Most current MPI scanners are cylinder-shaped and mainly suited for head imaging, which limits their clinical use. In this study, we tested our single-sided MPI scanner designed to image larger and harder-to-reach areas like the breast. Using an anatomical breast phantom with simulated point tumor sources without a priori known locations, the system successfully identified the tumors in multiple imaging planes, independently confirmed by ultrasound. The scanner was sensitive enough to detect as little as 4.0 μg of iron, well below the level estimated for successful breast tumor detection, and to image depths up to 3.5 cm. These results show that single-sided MPI could be a viable approach for breast imaging application and may be suitable for future clinical use.

## 1. Introduction

Breast cancer is the most prevalent cancer among women worldwide and in the United States [1]. The current standard screening method, X-ray mammography, has an overall sensitivity of around 80% [2]. However, its effectiveness significantly decreases for women with dense breast tissue, leading to higher rates of false positives and false negatives [3]. Additionally, mammography is associated with considerable patient discomfort. Other imaging modalities, such as magnetic resonance imaging (MRI), ultrasound, computed tomography (CT), and single-photon emission computed tomography (SPECT), used for molecular breast imaging (MBI), are available but are generally used as supplemental techniques due to limitations in sensitivity, cost, imaging time, and the use of ionizing radiation [4]. These challenges highlight the need for a novel screening approach that is safe, highly sensitive, cost-effective, and robust.

In addition to early detection, precise localization of malignant cells is crucial for effective breast cancer treatment. Breast-conserving surgery remains a preferred option, but complete tumor removal is essential to prevent recurrence. Therefore, intra-operative imaging techniques to delineate tumor margins are currently under development [5]. One example of an emerging method to distinguish normal from cancerous tissue is photoacoustic imaging. This non-invasive, high contrast imaging method visualizes differences in optical absorption using a pulsed laser to elicit an acoustic response from tissue [6]. Another experimental tracer-free modality that has been investigated for breast cancer detection is microwave imaging [7].

Magnetic Particle Imaging (MPI) is another emerging tracer-based biomedical imaging modality capable of detecting superparamagnetic iron oxide nanoparticles (SPIONs) with exceptional sensitivity, contrast, and high spatiotemporal resolution [8]. MPI is considered a safe imaging technique, as it does not involve ionizing radiation and utilizes non-toxic SPION tracers, some of which have been approved for use in magnetic resonance imaging (MRI) applications [9,10]. Similarly to MBI, MPI has the potential to distinguish between malignant and benign tumors through both passive and active tracer-targeting mechanisms [11]. Passive accumulation may result from the enhanced permeability and retention (EPR) effect, in which nanoparticles preferentially accumulate in tumors due to abnormal vasculature and impaired lymphatic drainage [12]. In addition, SPIONs can be functionalized with tumor-targeting agents that bind to biomarkers associated with malignant tissue [13]. These mechanisms can produce sufficient SPION accumulation for in vivo tumor imaging, particularly when nanoparticle size, coating, and circulation time are optimized. Multiple groups have demonstrated MPI detection of tumors in murine models following systemic SPION administration, including 4T1 tumors, which are commonly used as a model of triple-negative breast cancer [14,15,16]. Therefore, MPI shows potential as a supplemental approach for diagnostic imaging and tumor margin detection in breast cancer applications.

Over the past decade, MPI technology has advanced rapidly, leading to the development of commercial preclinical MPI systems with small-bore designs, such as those offered by Bruker (Karlsruhe, Germany) and Magnetic Insight (Alameda, CA, USA). The utility of MPI imaging was demonstrated in a range of preclinical applications, such as angiography [17,18], and perfusion [19]; cancer imaging [20], cell tracking [21,22], hyperthermia [23,24], and real-time tracking of interventional instruments: e.g., balloon catheters in cardiovascular procedures [25,26]. Significant research efforts are underway globally to develop human-sized MPI systems, with a primary focus on closed-bore, head-only scanners [27,28,29,30], and targeted applications in functional MPI [31] and cancer detection and treatment [32]. Recently, the utility of interventional procedures was demonstrated with the leg interventional scanner, iMPI [33,34]. Despite these advancements, the extension of MPI technology to whole-body imaging remains challenging due to safety concerns and the high power requirements associated with closed-bore geometries [35].

In this work, we explore the suitability of an MPI scanner with an asymmetric geometry for breast tumor imaging and localization. A single-sided MPI scanner has all hardware on one side [36,37,38], allowing unrestricted access to the imaging subject. Our scanner features several unique design elements [39,40], which provide superior sensitivity and image fidelity compared with other single-sided MPI systems. This topology enables imaging of small regions of interest in large animals or human subjects, making it a promising candidate for breast cancer imaging applications. Recent developments in SPION detectors include handheld probes, which can be helpful in various breast cancer-related applications such as sentinel lymph node (SLN) biopsy [41,42,43,44], and breast-conserving surgery [45,46]. Other promising alternatives include handheld and freehand MPI scanners that have demonstrated SPION detection for localization and intraoperative guidance [47,48]. Due to their low form factor and suitability for handheld operation, such systems operate with lower-power magnetic fields and greater magnetic field inhomogeneity, which may lead to lower sensitivity and reduced penetration depth. Consequently, a purely single-sided design is not a universal solution for whole-body imaging. However, integrating a magnetic field-free region in the form of a line referred to as a field-free line (FFL) gradient topology into a single-sided MPI scanner [49] could generate sufficient magnetic field gradients and excitation fields for imaging peripheral organs, including breast cancer screening, SLN imaging, and staging.

Our single-sided MPI scanner employs an FFL-based gradient field and a 25 kHz excitation field [39,50], leveraging safety standards [51], high spatial resolution, and the enhanced sensitivity of FFL technology, which has been shown to provide up to a 10-fold gain in sensitivity over an alternative field-free point (FFP) configuration of field-free region while enabling robust image reconstruction with a well-established technique [52], specifically filtered back projection (FBP). This scanner geometry enables imaging of anatomical targets that are difficult to accommodate within conventional closed-bore MPI systems. Here, we demonstrate this capability by imaging an anatomically realistic breast phantom containing two embedded spherical SPION tumor models. This subject was imaged in two separate planes: tomographic imaging in the coronal plane and 2D projection imaging in the axial plane. These experiments achieved in-plane spatial resolutions of 6 mm full width at half maximum (FWHM), demonstrating sub-centimeter performance. These results were comparable to those achieved in MBI [53]. Thus validating our single-sided MPI scanner’s ability to image larger objects beyond the capabilities of current commercial MPI systems while maintaining clinically relevant resolution.

## 2. Materials and Methods

### 2.1. Single-Sided FFL MPI Scanner

**Figure 1:**
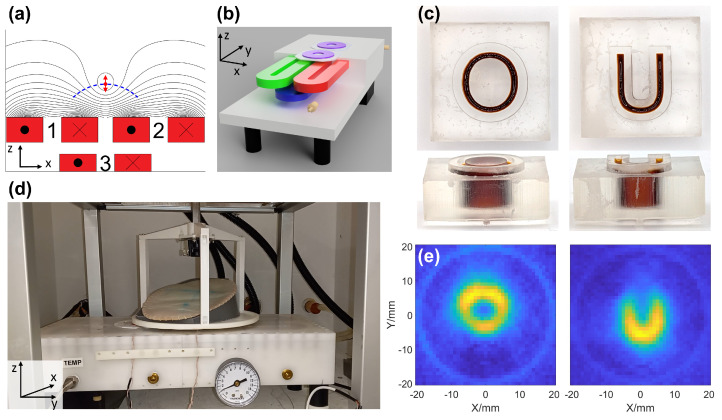
Setup of single-sided FFL scanner. (**a**) Cross-sectional schematic of the single-sided MPI scanner, showing internal coils and magnetic field geometry. Selection coils (1 and 2, red blocks) are positioned on the scanner surface, with the excitation coil (3) located below. Black lines represent the static magnetic field generated by the selection coils, forming an FFL in the center of the scanner (red dot). The dashed blue arc indicates the range of FFL positions achieved by varying the relative currents. Red arrows show the direction of the excitation field. (**b**) 3D rendering of the scanner assembly. Selection coils (red and green) near the scanner surface, with the excitation coil (blue) below them. The receive coil and gradiometer coils (purple) are located on the surface of the scanner. (**c**) Photograph of two SPION-filled phantoms shaped as the letters “O” and “U” shown from both top and orthogonal views. (**d**) Photograph of the experimental setup. A breast phantom is positioned inside a holder in the center of the sample table, which allows for rotation and vertical translation of the subject. (**e**) Examples of MPI images of “O” and “U” phantoms acquired with our single-sided scanner, demonstrating its ability to image complex geometries.

Our single-sided FFL MPI scanner is designed to facilitate imaging of near-surface structures, such as breast tissue. Unlike conventional MPI scanners with closed-bore geometries, the single-sided design allows for imaging large or irregularly shaped objects, at the cost of limited penetration depth. The system comprises three racetrack-shaped electromagnetic coils embedded in a custom-built water-cooled enclosure by Resonance Research Inc. (Billerica, MA, USA), as shown in Figure 1b,d, which generate the excitation and selection of magnetic fields required for imaging. Additionally, Figure 1c,e shows results of imaging of “OU” letter-shaped phantoms, illustrating typical phantom imaging with the scanner.

At the center of the scanner is the excitation coil, which produces an alternating magnetic field at a frequency of 25 kHz, referred to as the excitation field. This field is oriented along the z direction at the center of the FOV, and develops a small x component at lateral positions from the center. The direction of the excitation field is illustrated in Figure 1a with red arrows. Nonlinear oscillations are induced in the SPIONs by the excitation field and are detected by a circular receive coil located on the surface of the scanner. To suppress background signal originating from feedthrough with the excitation coil, an additional gradiometer circular coil, identical to the receive coil and functioning as a gradiometer, is positioned adjacent to it [54].

The selection coils (Figure 1b [red, green]) are located beneath the surface of the scanner, equidistant from its center. These coils generate a gradient magnetic field that creates an FFL. This FFL represents the region of sensitivity for SPION detection, as SPION particles located outside this region produce negligible signals. Generating the FFL and scanning its position allows for spatial encoding of our signal. Due to the single-sided geometry of the selection coils, the trajectory of the FFL represents an arc shown in Figure 1a as a blue dashed line. This trajectory, which has been described in [39], can be linearized by implementing a linearization algorithm as demonstrated in [40,55].

The imaging subject is positioned on an 18.5 cm diameter sample table (see Figure 1d) capable of rotation in the xy plane and vertical translation along the z-axis, allowing for mechanical depth encoding of the imaging volume. The imaging volume is located above the surface of the scanner, spanning a 4 cm × 4 cm plane located 17 mm above the device. Due to the single-sided nature of the scanner, the magnetic field strength varies with position, leading to spatially dependent sensitivity. However, within the 4 cm imaging region used in this study, the fields are sufficiently homogeneous that the resulting sensitivity is approximately uniform across the FOV. At the center of the scanner’s surface, the excitation field amplitude is 1.2 mT. For coronal imaging, the gradient strength is maintained at 0.58 T/m across the imaging plane to ensure uniformity, which is necessary for avoiding artifacts when utilizing filtered back projection (FBP) reconstruction. In axial imaging, where FBP reconstruction is not required, a non-uniform gradient is used, achieving a field strength of 0.84 T/m at the center of the imaging volume.

The single-sided MPI scanner uses low-noise electronics for both excitation and detection of the SPION signal. A 25 kHz excitation waveform generated by a DS360 function generator (Stanford Research Systems, Sunnyvale, CA, USA) is amplified using a Techron 7548 power amplifier (AE Techron, Elkhart, IN, USA) and routed through a custom low-pass filter with a cutoff near 25 kHz [39,40]. The resulting ∼6.8 $A_{peak}$ current generates an inhomogeneous excitation field, with an amplitude of 1.6 mT at the scanner surface and an amplitude of 0.78 mT at a height of 17 mm above the scanner, corresponding to the center of the FOV.

Signal detection is performed using a custom wound circular receive coil [54] consisting of 38 total turns of AWG 22 Litz wire (40/38), in two layers. The coil is wound on a 3D printed ABS frame with an inner diameter of 21 mm, and an outer diameter of 60 mm, with a total thickness of 4 mm (including base), and a measured inductance of 121 μH measured at 25 kHz. This coil is mounted on the surface of the scanner and is paired with an identical coil, which acts as a differential gradiometer, attenuating 41 dB of direct feedthrough. The differential signal is then routed to a commercial high-pass filter (EF125, Thorlabs, Newton, NJ, USA) with a 50 kHz cutoff, providing 46 dB attenuation at the 25 kHz fundamental frequency. The filtered signal is then input to an SR860 lock-in amplifier (Stanford Research Systems, Sunnyvale, CA, USA), which demodulates the amplitude of the third harmonic, the dominant harmonic after the fundamental. The measured third harmonic amplitude is transferred to a PC via a GPIB interface for storage and subsequent image reconstruction. The phase of the third harmonic is also measured, as it can provide information about SPION relaxation behavior. However, since all experiments used static phantoms with the same SPION tracer, phase differences are minimal and therefore not analyzed in this work.

### 2.2. Breast Phantom Details

**Figure 2:**
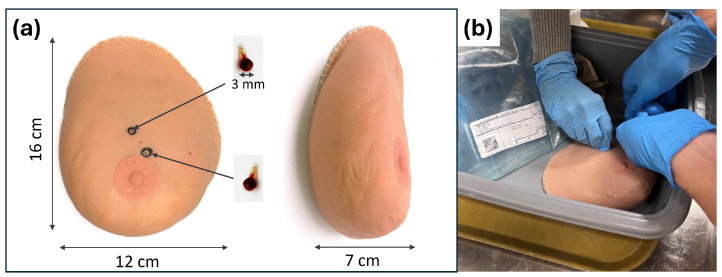
Anatomical breast phantom with implanted SPION tumor models. (**a**) Photographs of the anatomical breast phantom used in this study shown in two orthogonal views with labeled dimensions. The phantom measured approximately 16 cm in length, 12 cm in width, and 7 cm in thickness. Two artificial tumors consisting of 18 μL glass bulbs filled with Synomag-D SPION tracer were implanted 7 mm below the phantom surface. The tumors were positioned at the 12 and 1 o’clock locations, 39 mm and 20 mm from the nipple, respectively, and spaced 16 mm apart center-to-center. (**b**) Photograph showing the insertion of the glass bulb SPION tumor models into the breast phantom prior to imaging.

To validate our single-sided MPI scanner in accommodating the realistic human breast, we acquired an anatomical medical training breast phantom (SynDaver, Tampa, FL, USA). While its complex tissue structure can be visualized with conventional modalities such as ultrasound, MPI does not directly image tissue. Therefore, artificial SPION-labeled tumors were implanted into the phantom. Specifically, two small glass bulbs (18 μL each) were filled with undiluted PEG-coated Synomag-D 50 nm (MicroMod, Rostock, Germany) (10 mg/mL; Lot 20322104-02) SPIONs to serve as artificial tumors. These “tumors” were implanted 7 mm beneath the skin of the phantom and positioned at the 12 and 1 o’clock locations, 39 mm and 20 mm from the nipple and spaced 16 mm apart (center-to-center). The breast phantom with the implanted tumors can be seen in Figure 2a.

The breast phantom has realistic synthetic human tissue that mimics the mechanical, thermal, and physicochemical properties of live tissue. It consists of skin, subcutaneous fat, bulk fat, a natural wear layer of dead skin at the surface, and three discrete layers (epidermis, dermis, and hypodermis) that move independently from one another. The synthetic human tissues are made from salt, water, and fiber. The phantom internal structure is visible by the clinical standard imaging modalities such as ultrasound, MRI, CT, and X-ray.

To enable stable and repeatable positioning of the breast phantom during imaging, a custom-made holder was fabricated to conform to the phantom’s geometry. To construct this form, a 3D image of the breast phantom was acquired using the WIDAR 3D Scan & Edit app with Apple iPhone 13 (Apple, Cupertino, CA, USA), capturing a full $360^{{}^{\circ}}$ view of the object. The resulting mesh was imported into Fusion 360 (Autodesk, San Francisco, CA, USA) and used to generate a conformal mold by subtracting the breast phantom volume from a cylindrical shell. Producing a negative cavity stabilizes the phantom’s position throughout scanning.

The resulting holder design was exported as an STL file and fabricated with a filament 3D printer (QIDI I-Fast, Ruian, China). The holder was printed with a base thickness of approximately 2 mm to maximize usable FOV in the vertical direction of our FOV, while still preserving mechanical stability. The thin-wall structure allowed for consistent positioning of the phantom within the scanner’s FOV across multiple scans.

### 2.3. MPI Imaging Protocol

To acquire images with our MPI scanner, the FFL is scanned across the subject to generate a 1D projection of the SPION content. The FFL is discretely stepped by electronically varying the relative DC currents in the selection coils inside the scanner [39]. Scanning covers a 4 cm range, centered at the scanner’s midpoint. Due to the geometry of the selection coils, the FFL follows a fixed arc above the scanner surface, reaching a maximum height of 17 mm at the center and decreasing to 10 mm at the edges of the 4 cm FOV (±20 mm).

The process begins by forming the FFL at the leftmost position, −20 mm. At this initial position, a 203 ms delay ensures sufficient time for eddy currents to dissipate before signal acquisition. The third harmonic signal is then recorded for 70 ms. Subsequently, the FFL is incremented by 1 mm, followed by a shorter 47 ms stabilization delay, as smaller position changes require less settling time than the initial setup. The third harmonic is recorded again for 70 ms at each new position. This procedure is repeated for 41 FFL positions, spanning from −20 mm to 20 mm, with each complete scan of the 4 cm range taking 5 s. To improve the SNR of the 1D projection, the process is repeated for multiple scans and averaged. For imaging of the tumors, six averages were used per projection, except for fast coronal imaging, resulting in a total acquisition time of 30 s per projection.

To perform tomographic imaging in the XY plane of our scanner, multiple 1D projections of the sample are acquired at 20 different angles, resulting in a total imaging time of 10 min. After each 1D projection is measured, the sample is mechanically rotated by 9° using a custom-made rotation table. The FFL is then scanned across the subject again to obtain a projection at the new angle. This process is repeated for 20 angles, spanning 0° to 180°, to construct a sinogram of the subject. The sinogram is subsequently reconstructed into a 2D slice of the subject using FBP with a Hann filter. To ensure uniform spatial encoding, which is required for FBP reconstruction, the gradient is 0.58 T/m at every FFL position in the FOV. Without this homogeneity, the spatial assumptions underlying FBP are violated, resulting in non-trivial complex artifacts in the reconstructed image.

To obtain a 2D projection image of the subject in the XZ plane, the sample is not rotated but is instead mechanically raised using the sample table. Imaging begins with the table positioned closest to the scanner’s surface, with its bottom 6 mm above the scanner’s surface. A 1D projection of the subject is acquired at this initial height. The table is then raised in 0.5 mm increments, and a new 1D projection is measured at each height. This process is repeated for 21 heights, covering a 1 cm FOV in the z-direction, and requires a total imaging time of 10.5 min. The final 2D projection is a collection of 1D projections at different heights. This image needs no reconstruction and is analogous to an X-ray image, as it is not tomographic. Because this requires no reconstruction, spatial encoding does not require homogeneity. We take advantage of this by increasing the gradient to 0.84 T/m at the center of the FOV, where gradient generation is more power efficient.

### 2.4. Ultrasound Imaging Protocol

Ultrasound imaging was performed using the Clarius L7HD3 linear-array ultrasound transducer (Clarius Mobile Health, Vancouver, BC, Canada) connected to an Apple iPad Air (Apple, Cupertino, CA, USA) to acquire an axial image of the phantom. Prior to imaging, ultrasound gel was applied to the breast phantom and the ultrasound probe to ensure proper acoustic coupling, and a disposable latex cover was placed over the probe for sanitation. The probe was manually positioned on the breast phantom and scanned as in a clinical breast ultrasound exam.

With the Clarius mobile application, the ultrasound image was frozen and saved to the local device. The depth of the identified tumors from the skin surface and the distance between the two tumors were then measured from the saved ultrasound image and compared to the respective measurements from the MPI image.

## 3. Results

### 3.1. MPI Imaging of Breast Phantom

**Figure 3:**
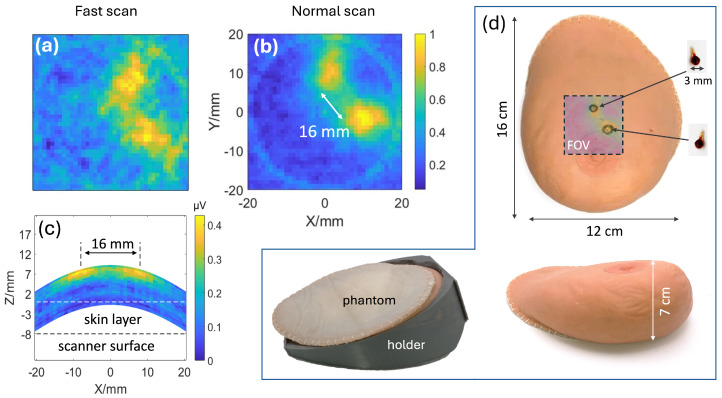
MPI images of Anatomical breast phantom. (**a**) MPI image of artificial SPION tumors from a fast scan (100 s) in a coronal plane. (**b**) High SNR MPI image in a coronal plane of tumors from a slow (10 min) scan. (**c**) MPI image of breast tumor model in axial plane (total acquisition time 10.5 min). (**d**) Anatomical breast phantom with labeled dimensions in two orthogonal views. Two 18 μL glass bulb SPION (Synomag-D 50 nm) tumors are embedded in the breast phantom 7 mm under the skin, placement shown as black circles, and the respective imaging FOV is indicated as a square. (**Bottom-left**): 3D printed holder for the breast phantom with the breast phantom placed inside in a prone position, used for consistent positioning of a phantom in the scanner.

To evaluate the ability of the scanner to image an anatomical breast-sized target, the phantom shown in Figure 3d was imaged using two complementary MPI imaging protocols designed to capture different views of the implanted SPION tumor models. The first protocol captured a 2D image in the coronal (XY) plane above the scanner. In this method, a pulse sequence was applied to acquire a 1D projection of the phantom, after which the phantom was mechanically rotated to capture projections at multiple angles. This process creates a sinogram that can be used to reconstruct a 2D image using FBP. To assess both clinical viability and scanner performance, two separate acquisition times were used. A short-duration scan (100 s) was used to demonstrate that viable imaging can be performed under clinically practical time frames, and a longer scan (10 min) was performed to obtain a high signal-to-noise ratio (SNR) image.

In the second protocol, the phantom was imaged in the axial XZ plane to form a projection image. Similarly, a pulse sequence was applied to obtain a 1D projection, but instead of rotation, the phantom was mechanically raised 0.5 mm, and the field was electronically shifted to different points to sweep across the axial slice. This creates a 2D projection of the SPIONs inside the imaged subject, with no reconstruction required. The total acquisition time for this sequence is 10.5 min.

The resulting images are shown in Figure 3. The coronal image reveals two distinct hotspots corresponding to the tumor models, with a measured separation of 16 mm, consistent with their known implant location. The in-plane spatial resolution was estimated by extracting a 1D profile through the peak of each hotspot and computing the FWHM of the resulting one-dimensional intensity profile, yielding approximately 6 mm. The longer-duration scan (Figure 3b) provides high-contrast images with clearly resolved tumors, while the shorter-duration scan (Figure 3a), though noisier, still allows both tumors to be distinctly resolved. The axial projection further confirms the lateral separation of the tumors and measures their depth of 7 mm below the surface of the skin. In both views, the tumors are clearly resolved with high contrast and no visible background signal from the surrounding tissue. This tracer-specific contrast represents a fundamental advantage of MPI over anatomical imaging such as ultrasound, which generates a background signal from all tissue rather than targeted regions. Additionally, when compared with established tracer-based modalities such as SPECT, MPI does not utilize ionizing radiation, making it relatively non-invasive.

### 3.2. Ultrasound Imaging of Breast Phantom

**Figure 4:**
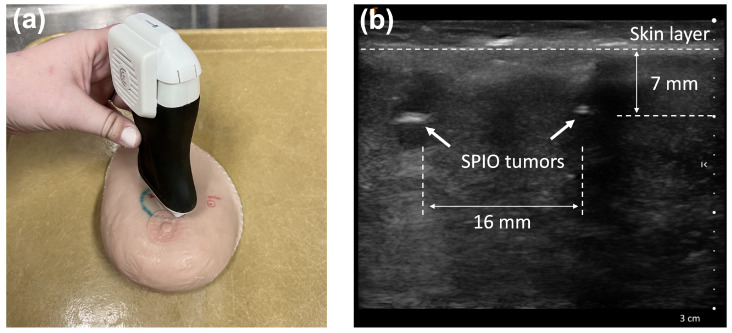
Ultrasound validation of SPION tumor positions in anatomical breast phantom. (**a**) Scanning with a handheld ultrasound imaging probe positioned on the top skin layer of the breast phantom. (**b**) Ultrasound image in axial plane showing two SPION tumors located 7 mm beneath the phantom’s surface, with a center-to-center separation of 16 mm. Dashed lines indicate the skin layer and horizontal positions of SPION tumors.

For reference to MPI imaging, ultrasound imaging was performed using a Clarius L7HD3 linear array (Clarius Mobile Health, Vancouver, BC, Canada), and the position of the tumors was measured. The ultrasound scan revealed two tumors, with the bottom lateral edge of the transducer 1 cm from the nipple and in an approximately radial position. The resulting measurements from the ultrasound images confirmed that the artificial tumors were located 7 mm beneath the breast phantom’s skin and were separated by 16 mm, as shown in Figure 4b. Both MPI and ultrasound imaging successfully detected the SPION-labeled tumors and allowed for the measurement of their relative positions within the phantom.

Due to the small size of the SPION tumor masses, they were difficult to fully visualize on ultrasound images. However, the bright reflection off the glass bulb confirms the presence of the SPION tumors. A dark artifact was observed beneath one of the tumors, likely caused by acoustic shadowing, which can occur when ultrasound waves are attenuated or reflected by dense or highly absorptive materials. Additionally, the inherent background signal from the phantom’s tissue-mimicking material reduced the contrast between the SPION tumors and the surrounding medium. While this limits the ability of ultrasound to directly visualize the tracer masses, it provides anatomical context that may be valuable when correlating ultrasound with MPI imaging.

### 3.3. Imaging Sensitivity: SPION Dilution Experiment

To evaluate the imaging sensitivity of our MPI scanner, we imaged a series of dilution phantoms in addition to the breast phantom. In this study, the scanner has been upgraded with a new excitation coil to enhance field uniformity across the FOV, a description of this excitation coil is provided in [40]. This coil provides a uniform 1.8 mT excitation field over the 4 cm FOV. The dilution phantoms consisted of the same 18 μL glass bulbs used as artificial tumors, filled with diluted PEG-coated Synomag-D 70 nm SPION (10 mg/mL, Lot 09324104-01) solutions. Notice that while the exact choice of SPIONs may matter for the dilution experiment, it doesn’t affect the undiluted breast phantom experiment. Each glass bulb was positioned at the center of the imaging FOV, 17 mm above the scanner’s surface.

**Figure 5:**
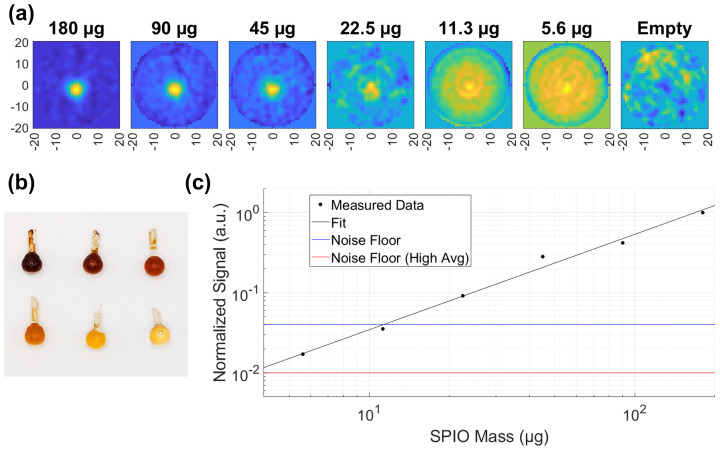
Sensitivity characterization using a dilution series of SPION-filled glass bulbs. (**a**) Coronal slices of MPI images of six glass bulb phantoms containing decreasing masses of SPION (180 to 5.6 μg). (**b**) Photograph of the dilution series, showing the corresponding glass bulbs arranged by SPION mass. (**c**) Signal plotted as a function of mass of solid SPION content on a log-log scale. The measured values show a linear relationship between the signal and iron content. The black line indicates the fit. Horizontal lines mark the noise floor (blue) and a lower noise floor for higher averaging conditions (red).

Six dilutions were prepared at 1:1, 1:2, 1:4, 1:8, 1:16, 1:32 ratios. Given the initial Synomag-D concentration of 10 mg/mL and a bulb volume of 18 μL, the corresponding solid contents of SPIONs were 180, 90, 45, 22.5, 11.3, 5.6 μg. The dilution phantoms are shown in Figure 5b. Imaging was performed with a 100 s acquisition time for all samples, except for the lowest concentrations (11.3 μg and 5.6 μg), which were averaged 16 times, resulting in a total scan time of 27 min. Noise floor measurements were computed as the standard deviation of pixel values in regions without SPIONs. Two separate noise floor values are reported, corresponding to the two separate acquisition times of 100 s and 27 min. If we assume particles consist of pure magnetite, then iron contributes to 72% of the total particle mass, and the lowest concentration would correspond to 4.0 μg of iron.

MPI images of the dilution phantoms and a plot of signal intensity versus dilution are presented in Figure 5a,c. This study demonstrates that our scanner can reliably detect iron masses as low as 4.0 μg with an SNR > 1.

### 3.4. Imaging Depth: Electronic Depth Experiment

To evaluate the ability of the scanner to image targets located deeper within tissue, a single SPION rod phantom was imaged at increasing distances from the scanner surface. The phantom consisted of a 22 mm long glass rod with an internal diameter of 1 mm filled with undiluted Synomag-D 70 nm SPION tracer (10 mg/mL). The rod was positioned at the center of the 4 cm imaging FOV and imaged at depths of 17, 20, 25, 30, and 35 mm from the scanner surface.

**Figure 6:**
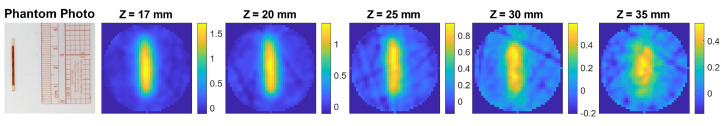
Electronic depth imaging experiment. A 22 mm long glass rod phantom with a 1 mm internal diameter, filled with undiluted Synomag-D SPION (10 mg/mL, 70 nm), was positioned at the center of the FOV and imaged at increasing distances from the scanner surface. Images are shown for depths of 17, 20, 25, 30, and 35 mm.

The rod was mechanically raised by the rotation table, shown in Figure 1d, to be positioned at different testing heights z relative to the scanner. For imaging, the FFL was scanned at the phantom height across a horizontal plane using the electronic height control algorithm discussed in [40,55]. The resulting images are shown in Figure 6. The SPION rod remained visible at all tested depths, including 35 mm from the scanner surface, although signal intensity and spatial resolution gradually decreased with increasing depth.

As the imaging plane is shifted farther from the scanner surface, both the receive coil sensitivity and the achievable field gradient decrease. The images at depths of 17, 20, and 25 mm were acquired with a gradient strength of 0.6 T/m, while the 30 and 35 mm images were acquired at reduced gradients of 0.51 and 0.33 T/m, respectively. This reduction in gradient strength results in decreased spatial resolution at deeper depths. In addition, the increased distance between the SPION rod and the receive coil reduces the detected signal intensity. These effects are visible in Figure 6, where the rod appears progressively wider, less sharply defined, and lower in intensity at greater imaging depths. Nevertheless, the SPION rod remained clearly visible at all tested depths. This experiment represents the first demonstration of electronic depth imaging with our scanner and demonstrates viable imaging at depths of at least 35 mm from the scanner surface, supporting the feasibility of imaging tumors located in deeper regions of the breast.

## 4. Discussion

In this work, we successfully demonstrated a proof-of-principle MPI imager capable of imaging an anatomical breast phantom with lifelike properties and a largest dimension of 16 cm. One of the primary advantages of the single-sided geometry is that it can accommodate objects of any size, even when they extend beyond the imaging FOV. Regions of interest can be brought into the scanner FOV by repositioning either the subject or the scanner. Reconstructed FBP images showed two distinct point sources with well-resolved separation, though not representing the system’s ultimate capability for spatial resolution [39]. Based on the point-spread function, the measured resolution in the coronal plane at the available gradient strength was 6 mm FWHM, demonstrating sub-centimeter imaging performance.

**Figure 7:**
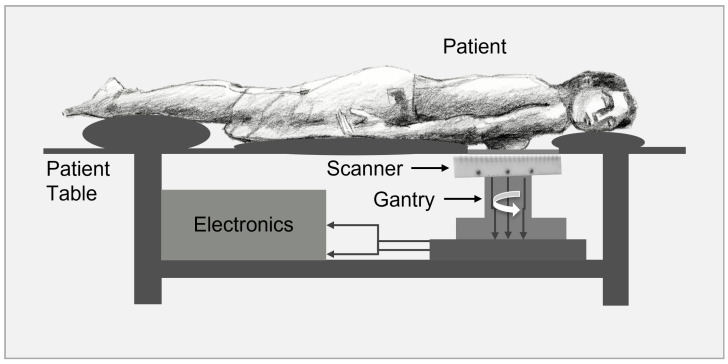
Conceptual design for a human-scale breast MPI scanner utilizing a single-sided FFL geometry. An integrated single-sided MPI scanner with a patient table, showing the patient comfortably lying in the prone position. The MPI scanner is mounted to a gantry system to allow for rotation of the scanner.

For potential clinical translation to be used as a diagnostic breast imager, several key improvements have to be implemented as depicted in the sketch in Figure 7 and are currently under investigation. First, the coil system has to be rotatable instead of the subject. Second, the imaging time of acquiring a slice has to be reduced to be within a minute. Third, the sensitivity has to be improved to allow less averaging time. Finally, the imaging depth should allow around 5 cm transversal FOV with sufficient gradient strength. Additionally, reproducible breast positioning could be achieved using a breast mold, potentially combined with optical 3D profile scanning. Co-registration with another imaging modality, such as X-ray or CT, could also provide additional anatomical guidance.

Previously, we demonstrated a sensitivity of our single-sided scanner up to 100 ng (Fe) in the MPI spectroscopy measurements using the same apparatus without image encoding [54]. To estimate the clinically relevant sensitivity, consider the average human injected dose (ID) of ∼400 mg (Fe) [56], the lower bound of a passive uptake of the SPIONs by the tumor of ∼1 (%ID)/g [57,58], then, for the average tumor mass of 1.2 mg per 1 ${\mathrm{mm}}^{3}$ voxel [59,60], the targeted sensitivity is 4.8 μg (Fe). Thus, the dilution imaging experiment (Section 3.3) shows that we are currently capable of imaging clinically relevant SPION concentrations. The future upgrades in coils and electronics would allow us to move closer to the ultimate 100 ng (Fe) sensitivity.

While MPI does not pose ionizing radiation risks, safety considerations are still necessary for human applications, primarily due to specific absorption rate (SAR) and magnetostimulation. These effects constrain the maximum excitation field amplitude in MPI. At frequencies below 42 kHz, magnetostimulation is the dominant safety concern, setting the excitation limit for full-torso imaging at 15 mT-pp for 25–50 kHz [51]. This threshold was estimated for full-torso imaging, and magnetostimulation limits scale inversely with object size $B_{lim}\propto \frac{1}{r_{obj}}$. Given an assumed breast radius of 80 mm (compared to a 20 cm torso radius), the magnetostimulation threshold for breast imaging is expected to be approximately 2.5 times higher, leading to an estimated limit of 37.5 mT-pp. Our scanner operates well within this limit, with an excitation field of 3.4 mT-pp, leaving room for increased sensitivity in future scanner designs.

The next generation of the scanner will be upgraded to incorporate a recently designed breast-specific surface excitation coil and to enhance the capabilities of selection field coils [40]. This would allow us to increase the sensitivity for detecting SPIONs and the FOV at least twofold, while simultaneously linearizing the encoding trajectory. In addition, the upgraded device will use a higher field gradient of up to 1 T/m, which would allow increasing the spatial resolution to 4 mm in the coronal plane.

As the next step towards breast imaging applications, we plan to validate our technique with in vivo imaging of breast tumor mouse models. Although cancer detection in rodent models was previously demonstrated using a small-bore rodent MPI scanner [20], to date, no in vivo MPI imaging has been demonstrated utilizing a single-sided MPI scanner that can accommodate large subjects.

## 5. Conclusions

In this work, we successfully imaged a breast phantom with implanted SPION tumors using our prototype single-sided MPI scanner. The results demonstrate clear separation and detection of two tumors separated by 16 mm, which was confirmed by ultrasound, validating the potential of MPI for breast cancer diagnostics. MPI offers distinct advantages over other imaging modalities, such as MRI or CT, by providing zero background signal from surrounding tissue, resulting in unambiguous high-contrast images. This capability is crucial for detecting small or diffuse tumor masses that might be missed with other techniques. Our unique single-sided design enables it to image phantoms larger than those accommodated by conventional closed-bore MPI scanners, making the topology well-suited for clinical applications involving human organs.

## Data Availability

The datasets generated during the current study are available from the corresponding author on reasonable request.

## Abbreviations

The following abbreviations are used in this manuscript:

ABS	Acrylonitrile Butadiene Styrene
AWG	American Wire Gauge
CT	Computed Tomography
DC	Direct Current
DL	Deep Learning
EPR	Enhanced Permeability and Retention
FBP	Filtered Back Projection
FFL	Field-Free Line
FFP	Field-Free Point
FOV	Field of View
FWHM	Full Width at Half Maximum
GPIB	General-Purpose Interface Bus
HPF	High-Pass Filter
ID	Injected Dose
LA	Lock-In Amplifier
LPF	Low-Pass Filter
MBI	Molecular Breast Imaging
MPI	Magnetic Particle Imaging
MRI	Magnetic Resonance Imaging
PA	Power Amplifier
PC	Personal Computer
Rx	Receive (coil)
SAR	Specific Absorption Rate
SC	Selection Coil
SLN	Sentinel Lymph Node
SNR	Signal-to-Noise Ratio
SPION	Superparamagnetic Iron Oxide Nanoparticle
Tx	Transmit (coil)
